# The Noble Suicide: The Case of a Self-Contained Dagger in the Heart and a Literal Raw

**DOI:** 10.1155/2024/3017903

**Published:** 2024-03-19

**Authors:** Maricla Marrone, Benedetta Pia De Luca, Marco Papalino, Fortunato Pititto, Carlo Angeletti, Roberto Bellacicco, Michela Raino, Giuseppe Pulin, Francesca Tarantino

**Affiliations:** ^1^Section of Legal Medicine, Interdisciplinary Department of Medicine, University of Bari Aldo Moro, 70124, Bari, Italy; ^2^Department of Mental Health, ASL Brindisi, Brindisi, Italy

## Abstract

According to WHO estimates, more than 700,000 people die each year due to suicide and suicides performed with a bladed weapon account for approximately 1.6%–3% of all suicides. It is statistically more common to find injuries to the heart, lungs, and thoracic vessels in homicides, whereas in suicides there is a higher frequency of vascular injuries to the extremities of the limbs. Also in suicides, the presence of “hesitation marks,” related to the attempts the victim makes before having the courage to kill himself, can often be found. In the case presented by the authors, these parameters are subverted: There was only one injury and it was the fatal one, it was located on the chest and reached the heart. But it was suicide. The circumstantial data, the psychological explanation, and the previous similar suicide attempt left no doubt about it. The man decided to commit suicide because he could no longer find meaning in his life after losing hope for a career as a pianist, having been diagnosed with a degenerative disease in his hands. The man hated himself and his existence: The future appeared extremely negative and the only escape was self-suppression. This case report makes an essential contribution to the already existing Literature as it shows a suicide that occurred in an unusual manner.

## 1. Introduction

Suicide by stabbing oneself in the heart is a gruesome and tragic method that is thankfully rare. While there are no official statistics on the number of suicides that involve this method, it is estimated that it accounts for a small percentage of all suicides.

The reasons for choosing this method of suicide are varied and complex. Some individuals may choose this method because they believe it will be quick and painless, while others may do so because of intense emotional distress or mental illness. Suicide by stabbing oneself in the heart may also be a final act of desperation for individuals who feel that they have exhausted all other options and can see no other way out of their situation [1, 2]. It is important to note that suicide is a complex issue and cannot be attributed to any one factor. It is often the result of a combination of factors, including mental illness, substance abuse, relationship problems, financial difficulties, and other life stressors. The act of stabbing oneself in the heart requires a significant level of determination and courage. It is not an easy task and requires a great deal of physical effort. As a result, it is not uncommon for those who attempt suicide in this manner to have made previous attempts or to have contemplated suicide for some time before attempting this method [1, 2]. There are many factors that can contribute to an individual's decision to take their own life in this manner. For some, it may be a response to overwhelming feelings of guilt, shame, or hopelessness. For others, it may be an attempt to escape physical or emotional pain. According to the Virgilian narrative of the Aeneid, Dido, Founder, and first queen of Carthage fell in love with the Trojan hero Aeneas when he landed in Carthage and they had an affair. Desperate for Aeneas' sudden departure, forced by Fate, Dido killed herself with Aeneas' sword [1, 2].

Jacopo Ortis (“Last Letters of Jacopo Ortis” 1802 novel of Italian literature), by “creation” of Ugo Foscolo, betrayed in his highest ideals, also planned his suicide. He said goodbye to his mother, wrote two letters, one to his beloved and one to a friend, and then took his own life by stabbing himself in the heart [3]. Edwin Shneidman's “Death Wish Theory” is a model that seeks to explain suicidal behavior through the concept of death wish and the inability to solve life's problems. It is based on the following concepts:

1. Shneidman emphasizes that the death wish, which can result from multiple factors (emotional, relationship, work, or financial problems) is a central component in the suicidal process. It represents the desire to escape an unbearable or painful situation.
2. According to Shneidman, the transition from the death wish to the suicidal act is influenced by the inability to solve life's problems.
3. Psychological stress is the key element in understanding suicide. The accumulation of emotional stress can contribute to the death wish and the inability to cope with life's challenges.
4. Stressful or traumatic events can trigger or intensify the death wish.

According to WHO estimates, more than 700,000 people die each year due to suicide [4]. According to the scientific literature, suicides carried out with a bladed weapon account for about 1.6%–3% of all suicides [5–7]. Most of these involve the use of kitchen knives or razor blades, and generally, the weapon used is found at the scene of the crime [8]. Self-inflicted injuries are located in most cases in the portions of the body that are easily self-aggressed with the dominant hand, namely the chest and upper extremities [9]. Numerous autopsy studies have shown that suicides involving stabbing choose bodily regions easily accessible with the upper limbs and those parts of the human body where the victim expects vital organs, damage to which would lead to a fast and relatively painless fatal outcome [10]. As a result, regions most likely to be involved in self-inflicted stabbings are the neck, the heart area, and the abdomen.

In cases of suicide by self-inflicted stabbing, the regions of the body targeted are typically the chest, abdomen, and neck. The chest region, specifically the heart, is often chosen due to the belief that it will result in a quick and relatively painless death. The heart is a vital organ that, when punctured, causes a loss of blood pressure and can quickly lead to unconsciousness and death. The abdomen region may also be targeted during self-inflicted stabbing, though it is typically considered a more painful and slower way to die. Puncturing the abdomen can result in damage to internal organs and lead to a slow and painful death by bleeding out. The neck region is another target for self-inflicted stabbing, though it is less common due to the risk of hitting major arteries and causing rapid and excessive blood loss.

The location of the self-inflicted injury can often take on special significance for the victim who follows a real “ritual” [11]. “Seppuku” is a Japanese term meaning “belly-cutting” and denotes an ancient ritual for compulsory or voluntary suicide [12]. It was performed, according to a rigidly codified ritual, as atonement for a fault committed or as a means of escaping a dishonorable death at the hands of enemies. The womb was believed to be the seat of the soul, and therefore the symbolic meaning underlying the ritual was to show the bystanders one's essence, free of guilt, and in all its purity. In the Edo period (1603–1867), it became a death sentence that did not involve dishonor: In fact, the condemned person was not executed but invited or forced to take his or her own life by practicing with a dagger a deep wound in the abdomen, severe enough to cause death [13].

The authors present the case report of a pianist who, following the diagnosis of an irreversible degenerative disease in his hands, decided to commit suicide with a kitchen knife, stabbing it into his chest just at the level of his heart. This case report makes an essential contribution to the already existing literature as it shows a suicide that occurred in an unusual manner.

## 2. Case Report

In November 2014, a 53-year-old man, around 6:30 a.m., while at his home with his spouse, struck himself in the chest with a kitchen knife.

**Figure 1:**
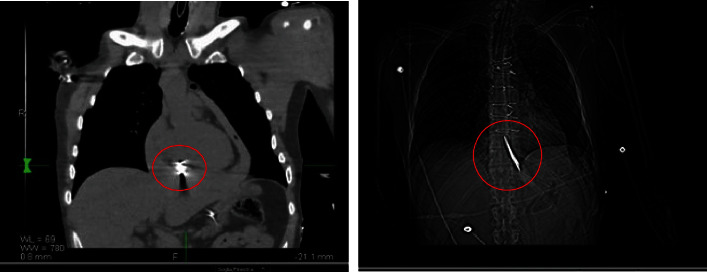
Frontal view of the chest on CT examination. The knife is represented by the red circle.

**Figure 2:**
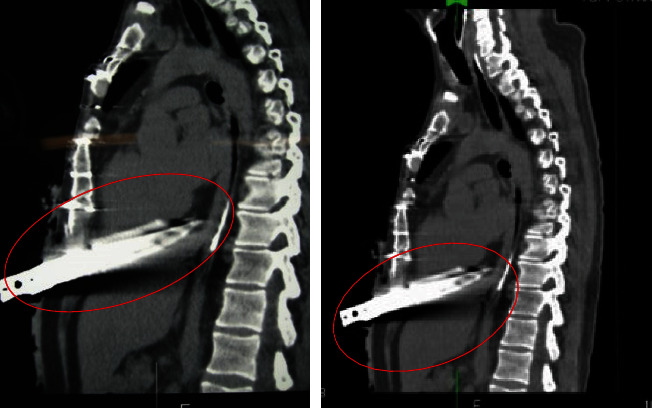
Lateral view of the chest on CT examination. The knife is represented by the red circle.

As a result of his injuries, Territorial Emergency Service 118 medical personnel were alerted and immediately transported him to the emergency room with a diagnosis of “state of shock with penetrating wound in epigastrium.” The ER sanitarians performed an infusion of four bags of red blood cells, placed the bladder catheter and venous access, intubated him, performed an urgent chest CT scan (images of which are shown in Figures 1 and 2), and alerted the surgical team to immediately transfer the patient to the operating room. However, despite the medical therapy promptly implemented by the medical team, at about 8:00 a.m., the patient passed away in the operating room.

From the testimony given by his mother, it was possible to deduce that he would always suffer from anxiety but he would also have started to suffer from severe depression after he was first diagnosed with a pathology in his hands that prevented him from performing his work as a pianist. Specifically, he suffered from Dupuytren's disease. Since then, the man was followed at the Mental Health Center with a diagnosis of “Major depressive disorder” and “Anxiety disorder” for which he allegedly took over time the following pharmacological treatment: sertraline, paroxetine, mirtazapine, and propranol. From the testimony given by the mother and from the psychiatric documentation collected, it was possible to reconstruct the clinical history of the man. He is described as an anxious man, who has always been particularly attentive to his health. In fact, he often turned to his primary physician to request investigations to rule out possible organic pathologies.

Just as a result of one of these checks, 4 years before the facts represented, he was diagnosed for the first time with a pathology of the hands. This pathology prevented him from performing his work as a pianist. In particular, he suffered from Dupuytren's disease.

Consequently to this discovery, the man introduced typical symptomatology of a “Disorder of the adaptation with anxiety and depressed mood.” Then, he turned to a psychiatrist specialist at the mental health center: He was advised to take Paroxetine first, with little benefit on symptomatology, and then Sertraline with slight improvement of the anxious picture and mood deflection. Specifically, the patient took Paroxetine at a dosage of 20 mg for about 1 month without having benefit on symptomatology. Instead, the same complained of anorgasmia as a side effect.

For that reason, the treatment was modified and he was advised to take Sertraline at a dosage of 50 mg. Such therapy yielded good benefit on anxiety symptomatology and deflated mood. During various follow-ups, however, periods, mostly brief and cyclical, of increased irritability, increased ideational flow, reduced sleep quality, nervousness, and distractibility were reported. During such periods, he was advised the concomitant use of benzodiazepines as needed for tranquilizing or hypnoinducing purposes. The mother also reported to us that he independently discontinued Sertraline therapy after about 16 months of treatment and shortly before the first suicide attempt that would later lead to hospitalization. As the disease progressed, man became aware of the concrete possibility of losing the ability to play in a short time. For this reason, about 2 years after diagnosis, the patient attempted suicide in the same way as this case report. On that first occasion, however, he was stopped in the act of committing suicide by his mother who accidentally intervened on the scene.

As a result of this incident, the patient was taken to the local emergency department. Then, he was admitted to the psychiatry department. Upon admission to psychiatry, the patient underwent Brief Psychiatric Rating Scale with a total score of 56 and rating of:

1. six for depression (item 3) and risk of suicide (item 4),
2. five for feelings of guilt (item 5), and
3. four for somatic concern (item 1) and anxiety (item 2).

No additional clinical scales administered to the patient are reported. At the time of admission, phases of increased irritability presented by the patient, with a cyclic pattern, and lasting more than 4 days with more than three concomitant symptoms including decreased sleep requirements, increased talkativeness, increased ideational flow, and increased agitation without; however, severe impairment of functioning were investigated in depth. After 7 days, he was discharged with diagnosis of “Bipolar Disorder II, severe depressive episode without mention of psychotic behavior” and treatment with lithium salts and Mirtazapine. Over the next 2 years, the patient presented substantial clinical stability. He continued to attend the mental health center and there would be no further admissions. A few weeks before the event, he decided to suspend drug treatment.

**Figure 3:**
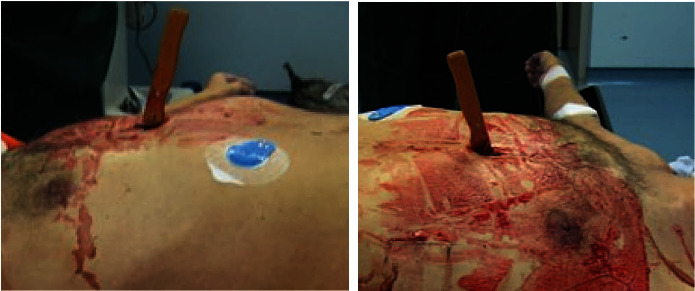
Left and right side view of the corpse.

The mother told us that, in the last period, the man showed particularly irascible and lamented greater difficulty in resting. Moreover, he presented the idea of ruin and guilt and had frequent crises of tears for the absence of hope for his future. As medical examiners, the authors were then alerted and went to the local P.S. There, they found the supine corpse on an operating table, covered with light blue sterile drape, with in place orotracheal tube attached to the assisted mechanical ventilation machine, as shown in Figure 3.

**Figure 4:**
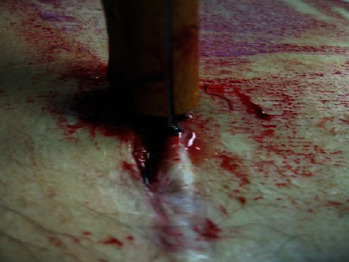
Continuous skin solution caused by kitchen knife.

At the middle region of the lower third of the anterior surface of the chest, there was the presence of a kitchen knife-type instrument penetrating into the torso with the back of the blade was facing upward, and the edge of the blade was facing downward, as shown in Figure 4.

When the knife was removed, it was found, just below the xiphoid process of the sternum, that there was a continuous solution of skin, triangular in shape, with apex pointing upward and base pointing downward, about 3 × 1 cm in size, with a major longitudinal axis. From the upper end (apex) of said lesion originated further superficial continuous solution of the skin, linear in longitudinal course, with a length of 1.5 cm, referable to “entrance tang”; we also detected, at the lower end (base), the presence of further superficial, linear, continuous skin solution, with a longitudinal course, 1 cm in length, also referable to “exit tang”; at the cautious specillation of said lesion, the authors noted that it was followed by an intracorporeal, blind-faced, oblique course, directed from the bottom to the top, and anteroposteriorly.

**Figure 5:**
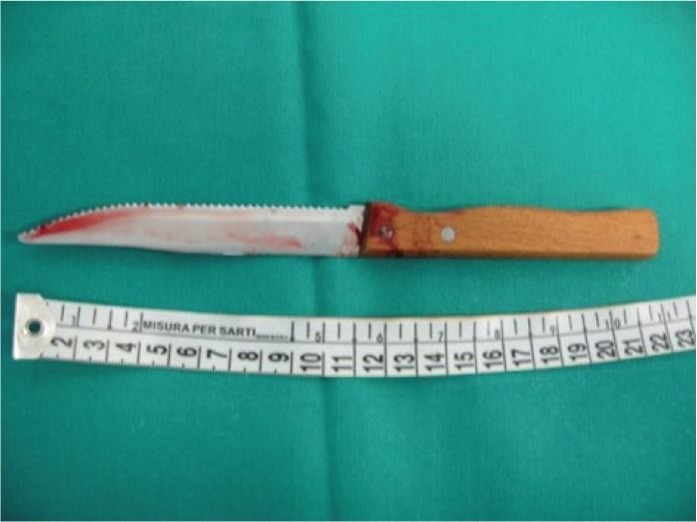
Medium of death: kitchen knife.

Analysis of the knife extracted from the corpse revealed that it was a white, single-edged weapon with an 11-cm-long blade, no serrations of the blade edge and a small blunt “serration” at the blade's attachment to the handle, a 0.2-cm-thick back of the blade, and a 10-cm-long wooden handle (Figure 5).

So, it was possible to trace the death to severe traumatic hemorrhagic shock resulting from severe cardiac injury, caused by a single-edged, 12-cm-long blade (kitchen knife).

In addition, taking into account, the injury in a self-aggressive zone (such as the chest) and the historical-circumstantial datum (previous suicide attempt with similar modalities, but no psychological or psychiatric treatment in place), it was possible to hypothesize a suicidal dynamic [5, 14]. It would appear that the man in order to fatally injure himself bent forward, and so much would justify not only the intracorporeal medium with a bottom-up direction but also the peculiar new “curved” body plan that would explain the inversion of the shanks (the long and thin entry one, the short and squat exit one) [15].

## 3. Discussion

Although suicidal blade injuries are found quite often during autopsies, suicidal stabbing is the rarest. In these cases, the main medicolegal problem in forced fatalities is the assessment of the manner of death. Forensic pathologists evaluate it primarily on the basis of clues and autopsy findings.

Self-inflicted stab wounds are generally multiple and, if so, are well laid out in parallel lines. In such instances, one larger wound is usually accompanied by several superficial stabs or cuts. If more than one deep fatal wound is encountered, it may point to homicide. In high-strength suicides, the weapon used must be found somewhere close to the body. The accompanying superficial injuries are known as tentative wounds (also hesitation marks or trial wounds) and are believed to be characteristic of a self-inflicted manner [16]. According to the literature, hesitation marks accompany more than half of cases of suicide by sharp tools [17].

The authors carried out a review of the literature and analyzed some cases similar to that presented in the manuscript (Table 1).

These wounds are produced voluntarily during a suicide while assessing pain and gathering the courage to inflict the final fatal wound. Although suicides with the absence of hesitation marks are possible, as it was documented in our case. In the case report reported by the authors, a patient suffering from depressive episode in bipolar disorder and anxiety is described.

Suicide is the leading cause of death in patients with bipolar disorder. In fact, that suicide rates among these patients are more than 20-fold higher than the general population and, furthermore, suicidal behavior is much more lethal in bipolar disorder than in the general population [25]. In addition, incorrect diagnosis and incorrect therapy increase the risk of suicide [26]. Depression is a complex clinical picture that varies over time and according to the individual [27]. The depressed person loses interest in everything around him or her, has a pessimistic view of life, no longer has joy in life, lacks concentration, often has fantasies of death, feels threatened by feelings of loss and “betrayed” by life [28, 29]. Most of the psychic energy is consumed in inner travail and the subject is thus “too tired” to enjoy the pleasures of life. The manifestation of the depressive state occurs through the feeling of deep disappointment from which a poignant loss of self-esteem is generated [30]. Depression, as pointed out by E. Weiss, has varying degrees of intensity [31, 32].

Bipolar disorder is a mental health condition that affects mood and behavior. Individuals with bipolar disorder experience episodes of both manic and depressive states, which can significantly impact their daily life. Unfortunately, bipolar disorder also carries a high risk of suicide. Studies have shown that individuals with bipolar disorder are at a greater risk of suicide than the general population. In fact, individuals with bipolar disorder have a suicide rate that is 10–30 times higher than the general population. This increased risk is due to a number of factors, including the intense and fluctuating moods that come with the disorder, as well as the impulsive behavior that can often accompany bipolar episodes. Individuals with bipolar disorder may also struggle with substance abuse, which can increase the risk of suicide even further. Substance abuse can worsen bipolar symptoms and lead to impulsive behaviors that may put the individual at risk of harm. Moreover, the depressed person experiences external stimuli with little interest, feels disinterest in affection, work, or any activity [33]. This is the stage of *melancholy*, in which the subject fails to put in place defense mechanisms to cope with such heavy inner despondency. The subject may consider his or her depressive status to be due to psychological wounds, the feeling of having fallen from grace, or from having been let down by someone [34, 35].

Depression becomes more complicated when the subject desires to “get rid” of his unbearable existential burden. The stage of *despair* begins, in which it is no longer possible to curb the internal self-destructive drives, partly because the depressed person often has a dissipation of interest from which his condition of despondency arises: The young man who kills himself after being ridiculed by his classmates overestimates the importance of the “offense” he received so much that he gives it more importance than his own existence [36]. The latter ineluctably loses value and can thus be suppressed. The melancholic in such cases plunges into apathy, the desperate person cannot bear “failure” and irrationally seeks a way to “erase it.” There is no more hope of “fixing” the impending situation; the only way left is to eliminate it [37]. Desperate Dido did not tolerate Aeneas' abandonment, desperate Jacopo Ortis could not bear the disappointment of his political ideals: Napoleon and the French had deluded Italy with their ideals of freedom, equality, and brotherhood and had in fact subjugated the homeland and ceded Venice to Austria. Both took their own lives finding no possible way out of their crushing disappointment. The desperate pianist could not tolerate the frustration of no longer being able to play because of the degenerative disease in his hands, and no hope could stop his intent to eliminate a now useless existence. Despair is the most advanced and furious state of despondency exposes him before an abyss, to a finish line that holds out no illusions. The only possibility the despairing person glimpses is to erase everything through death [38].

The depressed person goes from loving himself to hating himself irreparably. When the ego collapses due to adverse fortune the depressed person begins to hate himself to the point of punishing himself by killing himself.

Dido kills herself for love: The motivations that drive women to despair stem from the emotional context. It is the loss of prestige as “mother” or “lover,” disappointment in sentimental investment, and the disappearance of security within the family that generate despair. Ortis kills himself for the country, for the loss of ideals; the pianist kills himself for the loss of prestige as an artist and as a worker: It is not sentimental but careerist pride that interests the man; losing an important business, a career boost, can generate despair in the male. Despair will lead the depressed man to commit suicide, and the choice of mode delimits even better the characteristics of the gesture. The bladed weapon, whether a sword, knife, or dagger, in the literary imagination, represents the noble and swift death. Noble because such a weapon is the symbol of the valiant soldier, the means by which he fought in war, conquered territory, and defended the homeland. Swift because if thrust directly into the heart, the seat of “life,” it would with certainty lead to death, without waiting.

This mode (suicide with a dagger in the heart) also requires a great deal of courage on the part of the suicide (this is another reason why it was called a noble death): The perpetrator would not have to passively rely on an element of nature, such as water or the winds (perhaps by letting himself fall from a height), or on a helpless artifice such as a noose or poison, which would determine its own effect over time, but he had to wield a weapon with his own hands, turn it against himself and pierce himself.

## 4. Conclusions

Although most studies recognize this mode of death as resulting from homicide, the case presented by the authors tells the story of suicide by Major Depressive Disorder and Bipola Disorder II. Usually, in fact, sharp-force injuries inflicted to the head, chest, and back are more commonly found in homicides than in suicides, while injuries to the abdomen and extremities are more common in suicides. And so it is statistically more frequent to find injuries to the heart, lungs, and thoracic vessels in homicides, while in suicides, there is a higher frequency of vascular injuries to the extremities of the limbs. Also in suicides, the presence of hesitation marks can often be found, related to the attempts the victim makes before having the courage to kill himself [39].

All these elements were missing in the case presented by the authors: There was only one injury and it was the fatal one, it was located on the chest and reached the heart. But it was a suicide. The circumstantial data, the psychological explanation, and the previous similar suicide attempt left no doubt about it. The man decided to commit suicide because he could no longer find meaning in his life after losing hope for a career as a pianist. Vince hated himself and his existence: The future appeared extremely negative, and the only escape was self-suppression.

## Figures and Tables

**Table 1:** Case of literature.

	Gender	Age	Reason	
Case no. 1 [18]	Male	22 years old	The victim had a history of paranoid schizophrenia	A suicide caused by a single strong stab wound in the cardiac region
Case no. 2 [19]	Male	27 years old	The victim deceased had previously verbalized suicidal ideas and a suicidal message was recovered on his wife's phone	Planned complex suicide
Case no. 3 [20]	Male	—	—	Case of unplanned complex suicide with self-stabbing and head injury resulting from intentionally being struck by a train
Case no. 4 [21]	Male	19 years old	—	Case of complex suicide by self-stabbing and drowning
Case no. 5 [22]	Male	49 years old	—	Case of suicide by hanging in which multiple deep stab wounds were observed in the neck and chest
Case no. 6 [23]	Male	35 years old	6 years before death he had suffered from a severe cerebral trauma with intracranial hemorrhage, after which an organic psychosis had ensued	Case of suicide in which the victim was found dead within a vast collection of garbage in his parent's house with 13 stab wounds of the thorax
Case no. 7 [24]	Male	50 years old	—	Case of suicide by repeated self-stabbing on the chest with a sharp iron chisel
